# Preferences, Career Aspects, and Factors Influencing the Choice of Specialty by Medical Students and Interns in Saudi Arabia: A Cross-Sectional Study

**DOI:** 10.7759/cureus.43018

**Published:** 2023-08-06

**Authors:** Dana Sawan, Ghaday M Alrefaei, Abdulrahman Alesawi, Osamah Abualross, Shatha A Alsuwaida, Nuha Meer

**Affiliations:** 1 Obstetrics and Gynecology, Faculty of Medicine, King Abdulaziz University, Jeddah, SAU; 2 Medicine, Faculty of Medicine, King Abdulaziz University, Jeddah, SAU; 3 Medicine, University of Jeddah, Jeddah, SAU; 4 Medicine and Surgery, Faculty of Medicine, King Abdulaziz University, Jeddah, SAU

**Keywords:** saudi arabia, medical interns, medical students, medical specialties, future career, specialty choice

## Abstract

Background and objective

Choosing a medical specialty is one of the most critical career decisions medical students and interns make. However, little is known about the factors these graduates consider when choosing their specialty. Our study assessed factors that medical students and interns consider when determining their specialty.

Methods

This is a cross-sectional survey-based study, conducted from November to December 2022. We utilized a previously published questionnaire for 1074 participants, including 837 medical students and 237 interns from Saudi Arabian universities.

Results

The majority of female participants (80.4%), compared with only 19.6% of male participants, considered interest in specific procedures and techniques typical of the specialty an important factor in choosing a specialty (p = 0.036). Dissertation research experience was an important factor for 83.5% of female participants and 16.5% of male participants (p = 0.024). Additionally, good quality teaching within the study program framework was important for 81.2% of female participants and 18.8% of male participants (p = 0.033), suggesting that male and female participants viewed the importance of good quality teaching differently. Female participants accounted for 80% of those who considered the overseas experience a factor in their specialty choice. Also, 74.4% of female and 25.6% of male participants considered friends, relatives, or other connections in the healthcare field a factor that affects their choice. Furthermore, 79.6% of female and 20.4% of male participants reported having good experiences with physician role models as an impactful factor in their specialty choice.

Conclusion

Female participants were most interested in obstetrics and gynecology (12.1%,), internal medicine (11.8%), and family medicine (10.8%). Male participants, on the other hand, showed more interest in family medicine (12.7%,), internal medicine (11.0%), and emergency medicine (10.1%). Medical schools and healthcare institutions must provide students and interns with enough information and resources to help them explore different specialties and make well-informed decisions about their careers.

## Introduction

Selecting a medical specialty is an important decision that can have long-lasting effects on a future doctor's career. While personal interest is often a key factor in this decision, other considerations can come into play, such as job security, financial stability, gender, and familial circumstances [1].

For individuals pursuing a career in medicine, it is crucial that they thoroughly evaluate their options and make a well-informed decision regarding their specialty selection. This choice will have an impact on their daily work and their future employment opportunities, earning potential, and overall job fulfillment.

Choosing the right medical specialty will significantly affect a doctor's career trajectory and, therefore, requires careful consideration and guidance. This has been evidenced in studies conducted in Saudi Arabia that have investigated the factors that impact the choice of specialty made by medical students and compared them with findings from other countries. In one statewide study, career assurance, intellectual capacity, working with a diverse patient population, and financial stability were the primary factors influencing the decision-making of healthcare students [2]. Similarly, research conducted at the University of Dammam identified regional geography and lifestyle factors as significant determinants in selecting a specialty [3].

Medical students require access to career guidance and counseling services to make informed decisions about their future profession [4,5]. Similar results have been found in studies conducted in several countries. For example, a study in Pakistan discovered that the choice of specialty made by medical students was influenced mainly by personal interests, job security, and financial stability [6]. When choosing a surgical specialty, important considerations include patient care, outcomes, procedures, technical skills, and clinical issues. Stress was not a major factor for those pursuing a surgical career [6].

Although the determinants that affect specialty preference are generally similar across different countries, there are some differences resulting from country-specific factors and healthcare systems. For instance, in certain countries, gender and family influence can also be significant determinants [7].

There are other factors to take into account, including the type of procedures and technical skills required for a specific specialty, as well as the clinical problems encountered. Moreover, lifestyle factors and opportunities for career growth can also influence one's choice of specialty [8,9].

Choosing a medical specialty is a significant decision that can have a major impact on a doctor's career path. Several factors can influence medical students' choice of specialty, including personal interest, job security, financial stability, gender, and family circumstances. Understanding these factors is crucial for healthcare workforce planning, especially during times of medical worker shortages. However, there is limited research on specialty preferences and related factors among Saudi Arabian medical students. This study aims to determine the factors that impact specialty choice among Saudi Arabian medical students, including demographic, socioeconomic, and cultural factors. The study's findings could inform medical education programs and healthcare workforce planning efforts in Saudi Arabia, ultimately contributing to the provision of high-quality healthcare services and addressing potential workforce shortages in different medical specialties.

## Materials and methods

Study design and setting

This was a cross-sectional survey-based study utilizing a previously published questionnaire (see Appendix A). It was conducted from November to December 2022. The study was approved by the institutional review board at King Abdulaziz University in Jeddah, Saudi Arabia (approval no. 482-22). 

Participants

Our study involved 1074 participants, including 837 medical students and 237 interns from Saudi Arabian universities. We excluded persons who did not complete the survey and surveys with missing data. Participation in the study was voluntary and anonymous (no personal information was required).

Study procedure and questionnaire

An online self-administered survey about gender-specific factors involved in choosing a medical specialty was used to obtain participant data. Participants were randomly selected by sharing a link with medical students and interns. Before completing the questionnaire, the participants gave their consent to participate. The first section of the survey was to collect and categorize respondents according to sociodemographic characteristics. The second section inquired about their desired medical specialties upon completing medical school. The third section assessed important aspects of choosing a specialty, and the fourth assessed factors influencing their choice of medical specialty. Data were sorted using Google Forms (Alphabet Inc., Mountain View, CA, USA) and then exported for statistical analysis. 

Data analysis 

Data were analyzed utilizing SPSS Statistics version 29 (IBM Corp., Armonk, NY, USA). Proportions and frequencies were used to summarize categorical variables. Statistical analysis included examining the participants’ demographic characteristics, the medical specialties they desired, the aspects they considered important when selecting a medical specialty, and the key factors that influenced their choice of specialty. The chi-square test was used for bivariate analysis to determine statistical significance between the analyzed factors. A p-value less than 0.05 was considered significant.

## Results

The analysis included 1074 participants. Most of the participants were women (77.9%) and medical students (77.9%). The Makkah region showed the highest number of participants (39.0%). According to age distribution, most of the participants were between the ages of 20 and 25 years (80.2%) (Table 1).

**Table 1:** Demographic characteristics of participants (n = 1074)

Variables	Characteristics	Number	Percentage
Gender	Male	237	22.1%
	Female	837	77.9%
Age (years)	<20	55	5.1%
	20-25	861	80.2%
	26-30	125	11.6%
	31-35	20	1.9%
	>35	13	1.2%
Region of residence	Al-Baha	28	2.6%
	Al-Jouf	23	2.1%
	Asir	11	1.0%
	Eastern Province	87	8.1%
	Hail	11	1.0%
	Jazan	57	5.3%
	Madinah	172	16.0%
	Makkah	419	39.0%
	Najran	2	0.2%
	Northern Borders	3	0.3%
	Al-Qassim	54	5.0%
	Riyadh	71	6.6%
	Tabuk	136	12.7%
Current academic level	Medical intern	237	22.1%
	Medical student	837	77.9%

As seen in Table 2, female participants were most interested in obstetrics and gynecology (12.1%,), internal medicine (11.8%), and family medicine (10.8%). Male participants, showed more interest in family medicine (12.7%,), internal medicine (11.0%), and emergency medicine (10.1%). 

**Table 2:** Assessment of participants' desired medical specialties upon completing medical school (n = 1074)

Questionnaire	Male respondents		Female respondents	
Which specialty do you aim to pursue after graduation?	N	%	N	%
Adult critical care medicine	0	0.0%	2	0.2%
Anatomic pathology	1	0.4%	0	0.0%
Anesthesia	3	1.3%	17	2.0%
Cardiac surgery	8	3.4%	19	2.3%
Clinical biochemistry	0	0.0%	1	0.1%
Community medicine	0	0.0%	4	0.5%
Dermatology	6	2.5%	45	5.4%
Diagnostic radiology	7	3.0%	21	2.5%
Emergency medicine	24	10.1%	58	6.9%
Family medicine	30	12.7%	90	10.8%
Forensic medicine	3	1.3%	10	1.2%
General surgery	21	8.9%	57	6.8%
Internal medicine	26	11.0%	99	11.8%
Medical microbiology	0	0.0%	1	0.1%
Neurology	5	2.1%	23	2.7%
Neurosurgery	13	5.5%	24	2.9%
Obstetrics and gynecology	10	4.2%	101	12.1%
Ophthalmology	4	1.7%	29	3.5%
Orthopedic surgery	11	4.6%	14	1.7%
Otorhinolaryngology head and neck surgery	9	3.8%	36	4.3%
Pediatric medicine	12	5.1%	83	9.9%
Pediatric neurology	0	0.0%	4	0.5%
Pediatric surgery	6	2.5%	10	1.2%
Physical medicine and rehabilitation	0	0.0%	3	0.4%
Plastic surgery	5	2.1%	14	1.7%
Preventive medicine	0	0.0%	6	0.7%
Psychiatry	16	6.8%	47	5.6%
Radiation oncology	0	0.0%	6	0.7%
Urology	17	7.2%	13	1.6%

Table 3 presents career aspects participants considered important when choosing a specialty. The majority of female participants (80.4%), in comparison with only 19.6% of male participants, considered interest in specific procedures and techniques typical of the specialty an important factor, and this difference was statistically significant (p = 0.036). Results also indicate that career advancement opportunities and the possibility of working as a practicing physician were aspects the participants found important; however, the two factors showed no significant difference between male and female participants.

**Table 3:** Aspects considered important when choosing a medical specialty (n = 1074) * Statistical significance, p-value <0.05

Evaluative item	Response	Male		Female		p-value
		N.	%	N	%	
Advancement and career opportunities	Yes	101	22.1%	357	77.9%	0.992
	No	136	22.1%	480	77.9%	
Interest in specific procedures and techniques typical of the specialty	Yes	114	19.6%	467	80.4%	0.036^*^
	No	123	24.9%	370	75.1%	
Interest in the organs and pathologies specific to the discipline	Yes	91	19.6%	373	80.4%	0.091
	No	146	23.9%	464	76.1%	
Later earning opportunities	Yes	84	22.4%	291	77.6%	0.847
	No	153	21.9%	546	78.1%	
Reconciliation of family and career	Yes	65	20.4%	253	79.6%	0.404
	No	172	22.8%	584	77.2%	
Possibility of working as a practicing physician	Yes	78	20.2%	308	79.8%	0.271
	No	159	23.1%	529	76.9%	
Colleagues	Yes	107	21.6%	389	78.4%	0.717
	No	130	22.5%	448	77.5%	
Work atmosphere	Yes	107	21.6%	389	78.4%	0.717
	No	130	22.5%	448	77.5%	
Working hours	Yes	107	21.6%	389	78.4%	0.717
	No	130	22.5%	448	77.5%	

Table 4 presents various factors that influence participants’ choice of specialty based on their experiences and backgrounds. Dissertation research experience was reported as an important factor by 83.5% of female participants and 16.5% of male participants, revealing a statistically significant difference (p = 0.024). In addition, good quality teaching within the study program’s framework was reported as an important factor by 81.2% of female participants and 18.8% of male participants, with a statistically significant difference (p = 0.033) suggesting that male and female participants viewed the importance of good quality teaching differently. The participants also mentioned other factors. For example, female participants accounted for 80% of those who considered an overseas experience a factor influencing their choice of specialty, while male participants accounted for the remaining 20%. The table also shows that 74.4% of female participants and 25.6% of male participants considered friends, relatives, or other connections in the healthcare field as factors affecting their choice. Furthermore, 79.6% of female and 20.4% of male participants reported having good experiences with physician role models as an impactful factor in their choice of specialty (Table 4).

**Table 4:** Key factors influencing participants' choice of medical specialty (n = 1074) Chi-square test for bivariate analysis. *Statistical significance, p-value<0.05

Evaluative item	Response	Male		Female		p-value
		N	%	N	%	
Dissertation/research	No	200	23.5%	650	76.5%	0.024^*^
	Yes	37	16.5%	187	83.5%	
Experience abroad	No	174	22.9%	585	77.1%	0.293
	Yes	63	20.0%	252	80.0%	
Friends, relatives, etc.	No	182	21.2%	677	78.8%	0.165
	Yes	55	25.6%	160	74.4%	
Good experience through physician role models	No	139	23.4%	454	76.6%	0.228
	Yes	98	20.4%	383	79.6%	
Internship	No	126	20.5%	488	79.5%	0.158
	Yes	111	24.1%	349	75.9%	
Part-time job/previous healthcare training	No	194	22.9%	652	77.1%	0.188
	Yes	43	18.9%	185	81.1%	
Quality of teaching within the framework of the study program	No	155	24.3%	483	75.7%	0.033^*^
	Yes	82	18.8%	354	81.2%	

## Discussion

This study looked at medical students’ specialty preferences and factors influencing their choice of specialty, across all academic levels. Understanding these preferences is crucial for healthcare workforce planning, especially during medical worker shortages. While demographic preferences have been studied, specialty selection is influenced by complex factors, including lifestyle and sociodemographic influences [3,10-12]. The present study analyzed sociodemographic data of the study population, revealing a majority aged between 20 and 25 years and a higher proportion of female students. Most participants were single and in the clinical phase of their medical education.

Our study explored medical students' specialty preferences during their medical school years, after graduation, or during their internship. Among the latter group, internal medicine and family medicine were commonly desired specialties, while gender differences emerged in female participants preference for obstetrics and gynecology and male participants’ preference for emergency medicine. These findings align with previous studies highlighting the popularity of these specialties among medical students during this career stage [5,13,14].

Additionally, a study conducted by Compton et al. in the USA revealed that pediatrics and surgery were the most preferred specialties, contrasting with our findings [15]. Our study differs in terms of the least attractive specialties. While Compton et al. identified psychiatry and preventive medicine as the least attractive option, our study suggests that anesthesiology is the least preferred specialty. This difference could be attributed to the differences in the study population, methodologies, or regional preferences.

Family medicine is a preferred specialty among female participants in Saudi Arabia due to factors like shorter working hours and the need to address the underutilization of family physicians. However, the situation is different in North America, where family practice involves longer hours, heavier workloads, and greater public awareness [16].

Female students showed a preference for gynecology and pediatrics, consistent with previous studies [4,5]. Grasreiner et al. found a similar distribution of medical specialties among working physicians in Germany [17]. However, our study revealed that orthopedics and urology were preferred more by male than female participants, possibly due to the heavier workloads, on-call duty, and stress involved in these specialties.

Gender is a significant factor influencing the choice of specialty. A systematic review indicated that 63.7% of men were interested in radiology, while only 14.7% expressed interest in obstetrics and gynecology [18]. Another study with Saudi Arabian interns highlighted personal interest and workload, including on-call responsibilities, as major factors in specialty selection [2,19].

Significant gender differences were observed in participants' views on the importance of interest in specific procedures and techniques when choosing specialties (p = 0.036). Female participants represented the majority of those who value this factor (80.4%), compared with only 19.6% of male participants. However, career advancement opportunities and working as a practicing physician did not show significant gender differences.

Previous studies have highlighted various factors influencing the choice of specialty. Senf et al. emphasized income expectations, prestige, and breadth of knowledge as influential factors for selecting family medicine [20]. In our study, men showed a preference for the challenging specialty of emergency medicine. Zolaly et al. found that marital status played a major role for women, while personality and work achievement were significant factors for men [21]. Additionally, Eze et al. identified personal interest, career prospects, and personal skills/aptitude as influential factors in specialty selection [22].

Gender differences were also observed in the factors influencing the choice of specialty [1]. Dissertation research experience was important for 83.5% of women and 16.5% of men (p = 0.024), while good-quality teaching mattered to 81.2% of women and 18.8% of men (p = 0.033). Women were more inclined towards overseas experience (80% of women, 20% of men) and influence from health care connections (74.4% of women, 25.6% of men). Positive physician role models impacted 79.6% of women and 20.4% of men.

Both genders valued practicing abroad, research opportunities, perceived ability, and serving people. Men emphasized personal interest, family expectations, and future job prospects, while women prioritized teaching opportunities, work-related risk, and prestige [23]. Interestingly, men showed a preference for less competitive fields, potentially indicating gender differences in ambition and societal pressures [24]. Role models were an influential factor but did not rank highly. Previous studies support the positive impact of role models, including residents, although negative influences have also been reported. It is important to note that these findings are based on retrospective studies relying on the recollections of students [25,26].

Recommendations

This study provides insight into the preferences and factors that influence Saudi Arabian medical students when selecting their specialty. These findings should be taken into consideration when planning the healthcare workforce to address potential shortages in different fields. The study indicates that women tend to favor family medicine, while men show a greater interest in emergency medicine, internal medicine, and family medicine. The study also reveals gender differences in the factors that influence the choice of specialty, emphasizing the importance of considering these differences when designing medical education programs.

Limitations

The study has some limitations, which should be noted. First, it relied on an online survey with a convenience sample of medical students, which may only be representative of some of the population. Second, the sample was predominantly female, limiting the generalizability of the findings to include male medical students. Third, the study did not address potential regional differences in specialty preferences and factors influencing specialty choice. Finally, the study relied on self-reported data, which may be subject to recall bias. Nevertheless, the significant involvement of students from diverse medical colleges enhances the scientific significance of the data.

## Conclusions

This study shows that personal interest and other factors, such as job security, financial stability, gender, and family circumstances, can significantly impact medical students' choice of specialty. It also identifies gender differences in the factors that affect the choice of specialty, highlighting the importance of considering these differences in medical education programs. The findings emphasize the significance of career guidance and counseling services for medical students and interns to help them make informed decisions about their future professions. 

## Appendices

Appendix A 

**Table 5:** Questionnaire administered to participants

Questions	Multiple choice answers
What is your current academic level?	Medical student
	Medical intern
What is your gender?	Male
	Female
How old are you? (Years)	<20
	20-25
	26-30
	31-35
	>35
Which region in Saudi Arabia do you live in?	Al-Baha
	Al-Jouf
	Asir
	Eastern Province
	Hail
	Jazan
	Madinah
	Makkah
	Najran
	Northern Borders
	Al-Qassim
	Riyadh
	Tabuk
Which specialty do you aim to pursue after graduation?	Adult critical care medicine
	Anatomic pathology
	Anesthesia
	Cardiac surgery
	Clinical biochemistry
	Community medicine
	Dermatology
	Diagnostic radiology
	Emergency medicine
	Family medicine
	Forensic medicine
	General surgery
	Internal medicine
	Medical microbiology
	Neurology
	Neurosurgery
	Obstetrics and gynecology
	Ophthalmology
	Orthopedic surgery
	Otorhinolaryngology head and neck surgery
	Pediatric medicine
	Pediatric neurology
	Pediatric surgery
	Physical medicine and rehabilitation
	Plastic surgery
	Preventive medicine
	Psychiatry
	Radiation oncology
	Urology
What aspects are particularly important to you when choosing your specialty? (Multiple answers possible)	Advancement and career opportunities
	Interest in specific procedures and techniques typical of the specialty
	Interest in the organs and pathologies specific to the discipline
	Later earning opportunities
	Reconciliation of family and career
	Possibility of working as a practicing physician
	Colleagues
	Work atmosphere
	Working hours
What is the key factor influencing your choice of specialty in medical school? (Multiple answers possible)	Dissertation/research
	Experience abroad
	Friends, relatives, etc.
	Good experience through physician role models
	Internship
	Part-time job/previous healthcare training
	Quality of teaching within the framework of the study program
